# Identifying drivers of dengue fever outbreaks in Mauritius using Geographic Information System

**DOI:** 10.4102/jamba.v17i2.1740

**Published:** 2025-08-20

**Authors:** Smita Goorah, Manta Nowbuth, Mahendra Gooroochurn

**Affiliations:** 1 Faculty of Engineering, University of Mauritius, Reduit, Mauritius

**Keywords:** mosquito-borne diseases, Geographic Information System, multiple regression analysis, public health resilience, dengue fever

## Abstract

Mosquito-borne diseases can cause public health disasters. Climatic and environmental conditions, urbanisation, changes in land use, and the increased movement of people and goods worldwide are causing their increased transmission. Mauritius is especially at risk being situated in a vulnerable geographical region. In this study, we used geographical tools to identify potential drivers and vulnerability areas related to dengue fever in the island. Dengue cases were identified by municipal ward (MW) and village council area (VCA). Meteorological data consisted of rainfall and temperature data. The Relative Development Index (RDI) was used as a proxy for socioeconomic factors. The population density and the number of houses in close proximity to rivers were determined per VCA and MW. Maps were generated on the software QGIS 3.12. Statistical tests consisted of multiple regression analysis with dengue incidence as the dependent variable and potential drivers as the independent variables. The results showed that the close proximity of houses to rivers had a significant positive effect on dengue incidence (*p* = 0.03) while the RDI had a significant negative effect (*p* = 0.01). Vulnerability areas in the island can hence be determined based on the findings.

**Contribution:**

The findings of this study allow preemptive measures to be taken in identified vulnerability areas to prevent mosquito-borne disease outbreaks.

## Introduction

Mosquitoes are responsible for spreading severe diseases like malaria, dengue fever, chikungunya fever, Zika virus disease and West Nile fever among many others, causing public health disasters worldwide (World Health Organization [WHO] 2020). Mosquito-borne diseases (MBDs) are transmitted through blood meals. When a mosquito consumes the blood of an infected host, it also ingests the pathogens present. In a subsequent event, when the mosquito bites another person, it inoculates the pathogens into the new host causing infection (Gullan & Cranston 2005). The global burden of MBDs is illustrated by its considerable mortality. According to WHO estimates, malaria affects 219 million people in the world causing more than 400 000 deaths annually especially in children. Dengue fever is contracted by 96m people, and it causes 40 000 deaths annually. Billions of people in the world are extremely vulnerable to contracting an MBD (WHO 2020). With global warming and climate change predicted to increase MBDs, this situation is especially alarming, and global efforts are required to mitigate public health disasters.

## Literature review

Previously restricted to tropical countries, mosquitoes have been expanding their geographical range into temperate zones, and this trend is amplifying because of climatic changes (Samy et al. 2016). Moreover, the global trade of goods and the frequency of international travel have intensified the movement of infected mosquitoes and people, resulting in severe outbreaks across the world (Kilpatrick & Randolph 2012). Rigorous surveillance and rapid interventions are essential to prevent explosive outbreaks.

Rainfall is a major environmental driver, which regulates the reproduction cycles of mosquitoes. As there are obligatory aquatic stages in mosquito development, water is a critical element. Studies have shown that an increase in rainfall generates a greater availability of larval habitats. Therefore, more larvae are produced, leading to a rise in mosquito abundance (Manyi, Akaahan & Azua 2015). This is especially marked if there is accumulation and stagnation of water in puddles, waterlogged areas and in artificial containers among others. Rainfall also promotes the proliferation of mosquito species that prefer to lay their eggs in dry places. These mosquitoes choose to breed in transient environments rather than in permanent water bodies, and there is an expansion of their population after water levels rise (Calzolari 2016).

In addition to the amount, duration and time lag of rainfall, the frequency and the spacing of rainfall events are also relevant to mosquito abundance. It has been suggested that moderate to heavy precipitation increases near-surface humidity levels, which promotes egg-laying and host-seeking behaviour (Shaman & Day 2007). Hence, repeated rainfall events can increase disease transmission by influencing the mosquito’s reproductive activity and causing more breeding cycles. A study demonstrated that rainfall frequency occurring near the natural frequency of the mosquito reproductive cycle optimised the mosquito population growth and increased the transmission rates of MBDs (Shaman & Day 2007). This is an important result, as global warming may influence the frequency of rainfall events.

Mosquitoes are ectotherms. This means that they cannot generate body heat and are dependent on external sources of energy to maintain their body temperature at optimal levels (Gullan & Cranston 2000). The following events are heat-driven: egg viability, larval development, blood-feeding behaviour, female fecundity, adult longevity, interactions with parasites and arboviruses, wing size and population sizes (Ezeakacha & Yee 2019). Mosquitoes’ activity levels are also influenced by the ambient temperature, as they are more active in warm weather and more lethargic in the cold. Female mosquitoes also bite more frequently at higher temperatures, thus resulting in a higher risk of disease transmission (Githeko et al. 2000; Gullan & Cranston 2000). The environmental temperature also determines the mosquito distribution throughout the world. Warmer climates enable mosquitoes to increase their spatial distributions (Siraj et al. 2014), and this is expected to accelerate with global warming. In addition, with rapid urbanisation, human environments become warmer because of the Urban Heat Island effect and this promotes mosquito abundance (Misslin et al. 2016).

Microgeographic factors influence the prevalence of MBDs within short distances. These variables can modify microclimates, thus altering the abundance of mosquitoes in limited areas. The presence of freshwater sources, streams and other small water bodies favour mosquito proliferation. Local wind patterns affect mosquito movement and dispersal. Slight changes in elevations or variations in terrain or changes in light or shade can alter microhabitats and promote or hinder mosquito abundance. Landscape factors determine land use patterns such as urban settlements and agricultural practices. These exert an influence on the intensity of human–mosquito contacts. Hence, microgeographic factors are very important in understanding why some zones are more affected by MBDs than others are.

*Aedes albopictus* is currently considered to be one of the most invasive mosquito species. It is reported to be present in Asia, Africa, Europe, Australia, North and South America, and in Pacific and Indian Ocean islands including Mauritius. This is a remarkable global expansion considering its remote origins in the forests of South East Asia (Bonizzoni et al. 2013; Paupy et al. 2009). The *Aedes albopictus* species has the ability to survive in the cold as its eggs become dormant at low temperatures. This cold adaptation has enabled this species to extend to the chilly northern areas of the world. In the past, *Aedes albopictus* usually reproduced in natural habitats such as holes in trees, bamboo stumps and plants. As the species has expanded its habitats, it has modified its breeding habits to make use of a vast range of artificial containers present abundantly in environments modified by humans (Bonizzoni et al. 2013). The adult mosquito abundance is dependent on the seasons. Higher environmental temperatures and rainy weather are associated with mosquito proliferation as the larval development is stimulated (ECDC 2023).

Dengue fever has affected humans since ancient times. Its main clinical features include fever, headaches, rash, body aches, nausea and vomiting, mild haemorrhagic manifestations and mucosal bleeding (CDC 2023). Dengue fever became a global health issue because of the societal and ecologic disturbance caused by the Second World War in South-East Asia and Pacific regions. Unplanned urbanisation in tropical areas, global shipping and trade and worldwide movement of people by air travel to endemic tropical countries combined with the expansion of the *Aedes* mosquito vector into new territories are all causative factors for the worldwide diffusion of the disease (Gubler 1998).

Dengue fever manifested in the Indian Ocean islands in the 1940s. Initially, there were sporadic outbreaks in some islands. However, there is now an increasing incidence of dengue cases every year, with frequent outbreaks and co-circulation of different dengue virus serotypes in the Indian Ocean region. In addition, there have been recent reports of a shift from sporadic cases to endemic infections in the neighbouring island of Reunion (Hafsia et al. 2022). This continuous circulation of dengue viruses in close proximity to Mauritius coupled with the local presence of competent mosquito vectors is an alarming situation. Moreover, the island is well connected globally via dense airline networks to Africa, Asia and Europe. There is also a large influx of tourists and returning citizens entering the island and who may potentially introduce disease in the country. The high population density, rapid urbanisation, changing land-use patterns and existing population of mosquito vectors may cause rapid disease dispersion. Thus, the potential for mosquito-borne outbreaks exists, and there is a need for public health preparedness.

Geographic Information System (GIS) has been used to understand the spatial distribution of dengue fever. A study in Guangzhou, China investigated potential dengue risk factors of the 2014 outbreak by studying socio-ecological factors at a fine-scale spatial resolution involving small geographic units (Cao et al. 2017). Results indicated that temperature had the greatest influence on the incidence of dengue fever while rainfall was the second most important driving factor at the individual level. In addition, the interaction of high precipitation, high temperature, and high road density was found to increase dengue fever incidence. Another study in China also examined determinants of the 2014 dengue fever outbreak in Guangzhou (Chen et al. 2019). Geographical analysis was carried out using GIS software to study the effect of human and land-use factors. The study area was divided into a 1 km × 1 km grid to reflect the maximum mosquito flight range. The coordinates of dengue cases and the data about the five potential driving factors namely population, community age, subway, roads and ponds were embedded into each grid. Results demonstrated that the following human and land-use factors were significant: the population density, community age (ageing buildings and elderly people), subway network density and road network density (through their contribution to disseminating the disease) and ponds with standing water providing breeding sites. A study in Colombia investigated the contribution of socioeconomic and environmental factors to a 2010 dengue outbreak in Cali, Colombia by using a Geographically Weighted Regression model. Results indicated that population density, socioeconomic status and environmental factors such as the proximity to tyre shops and plant nurseries influenced disease risk (Delmelle et al. 2016). Satellite imagery can also be used in GIS to identify landscape characteristics that contribute to disease. A study carried out in Malaysia (Dom et al. 2013) used digitised satellite imagery to create a land-use layer. In addition, the housing type was determined and classified. The dengue cases for the year 2010 were geolocalised and superimposed on the above layers. Regarding housing, results showed that interconnection houses were the main risk factors for the spread of dengue fever (Dom et al. 2013). A study carried out in Quezon City, Philippines used a GIS approach to investigate spatial factors, which were associated with increased dengue fever cases in specific localities (Garcia & De las Llagas 2011). Dengue cases were geolocalised at the barangay level, which is the smallest administrative level. The following geographical attributes were identified to be associated with a higher incidence of dengue fever: zones with increased river networks, areas with built-up structures, residential lands with neighbouring commercial or industrial structures, and areas near dumpsite facilities mostly inhabited by the urban poor. On the other hand, increased population density alone was not directly associated with increased dengue fever cases (Garcia & De las Llagas 2011).

Thus, GIS tools are useful to determine the spatial risk of MBDs. They can be used to map the spatiotemporal patterns of disease; to identify risk factors such as landscape, socioeconomic, environmental and climatic determinants, and finally to locate those spaces where people are most vulnerable to disease.

This study aims to determine the potential drivers of MBDs such as dengue fever in Mauritius *using GIS*. In addition to identifying contributory factors, prior localisation of vulnerability areas in the island will guide preventive actions pre-outbreak and swift measures during an outbreak. The forecasting of potential outbreaks will also facilitate strategic control measures.

## Research methods and design

The island of Mauritius is divided administratively into village council areas (VCAs) and municipal wards (MWs). Shapefiles for the QGIS software have been constructed from data obtained from the Ministry of Housing and Land Use Planning (Mauritius), from Statistics Mauritius and from data that have been made publicly available, for example, United Nations Office for the Coordination of Humanitarian Affairs.

Dengue outbreaks in Mauritius were analysed geographically. Dengue cases in the public domain in the period 2009–2023 were compiled from public sources and from personal archives. There were no confirmed dengue cases prior to 2009. The cumulative dengue incidence (per 10 000 of population) was then calculated for the period by dividing the cumulative cases in each VCA and MW by the respective population and multiplying by 10 000.

Population data were obtained for the 160 VCA and MW using the 2022 census data (Statistics Mauritius 2022). The population density per km^2^ was calculated by dividing the population data by the relevant VCA and MW area. The number of houses situated close to rivers was obtained per VCA and MW (Statistics Mauritius 2022). The Relative Development Index (RDI) was obtained from the 2011 census data and was mapped by VCA and MW (Statistics Mauritius 2011).

According to Statistics Mauritius (2002):

[*T*]he RDI is a composite index that measures the relative achievement of sub-regions of the country in dimensions of development. It is the average of the following 12 variables used in the computation of the index are: % of households having piped water; % of households having electricity; % of households having flush toilet; % of households living in dwellings made of concrete; % of households having one or more rooms used for living purposes per person; % of households who own their dwelling; % of population aged 18 years and over having at least School Certificate or equivalent educational attainment; primary enrolment ratio; secondary enrolment ratio; literacy rate of the population aged 12 years and over; employment rate of the population aged 12 years and over; % of the employed population in occupational groups 1,2 and 3 of the International Standard Classification of Occupations (ISCO), i.e. legislators, senior officials and managers; professionals; and technicians and associate professionals. (Statistics Mauritius 2002)

Meteorological data consisted of rainfall data and temperature data. Rainfall data were obtained from the Mauritius Meteorological Services (MMS). The data consisted of long-term mean (LTM) rainfall per station per month of the year for 213 stations. The total annual LTM rainfall per station was interpolated, and a map was generated. The maximal rainfall data per VCA and MW were then estimated. Temperature data were also obtained from the MMS. The mean minimum temperature data for the month of February were estimated per VCA and MW. This month was selected as most outbreaks occur in February.

All maps were generated on the free software QGIS 3.12 obtained from the QGIS website.

The QGIS software is open-source and available for free download. It runs on multiple platforms. It provides powerful tools including data editing, analysis and cartography. In QGIS, layers are the fundamental way to organise, visualise and display spatial data on a map. Each layer corresponds to a specific dataset and defines how that data are visualised. Layers can be raster, vector or mesh, and they can be saved in various formats. Raster layers consist of pixel-based images, while vector layers represent data using points, lines and polygons. In this study, the layers consisted of shapefiles for VCAs and MWs, cumulative dengue incidence, population density, RDI, the number of houses situated close to rivers per VCA and MW and meteorological data.

### Ethical considerations

This study used secondary data available in the public domain.

## Results

### Overview of mosquito-borne diseases in Mauritius

Table 1 shows the evolution of MBDs in Mauritius since 1865. Malaria was the main MBD in historical times. Malaria also affected the island in recent times during the 1975–1989 period, in 1992 and finally in 1996 when the last outbreak was noted. Subsequent occurrences of MBDs involved a limited chikungunya outbreak in 2005 followed by an explosive outbreak in 2006. Recent years have seen several small outbreaks of dengue fever as illustrated in Table 1.

**TABLE 1:** Evolution of MBDs in Mauritius since 1865.

Outbreak	Year	Malaria	Chikungunya	Dengue
1	1865–1868	Yes	-	-
2	1908–1951	Yes	-	-
3	1958–1962	Yes	-	-
4	1975–1989	Yes	-	-
5	1992	Yes	-	-
6	1996	Yes	-	-
7	2005	-	Yes	-
8	2006	-	Yes	-
9	2009	-	-	Yes
10	2014	-	-	Yes
11	2015	-	-	Yes
12	2016	-	-	Yes
13	2019	-	-	Yes
14	2020	-	-	Yes
15	2023	-	-	Yes

### Distribution of population density by village council area and municipal ward

**FIGURE 1:**
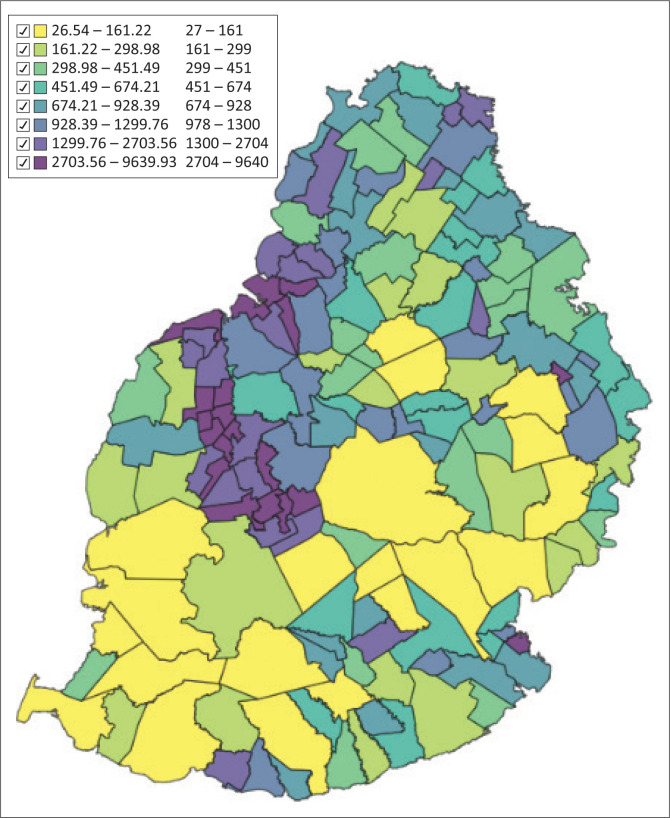
Distribution of population density by village council area and municipal ward (inhabitants/km^2^).

The distribution of population density by VCA and MW is shown in Figure 1. The population density is highest in urban areas MWs.

### Distribution of houses in proximity to rivers by village council area and municipal ward

**FIGURE 2:**
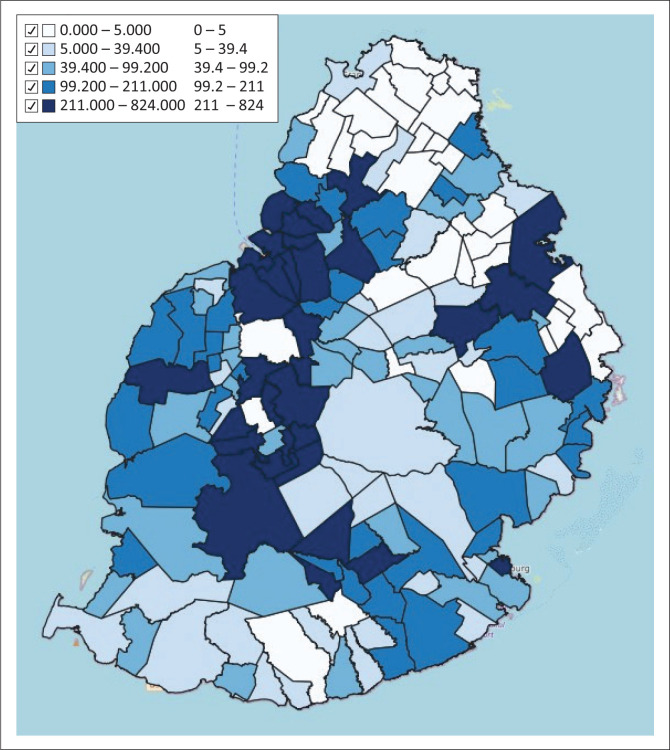
Distribution of houses in proximity to rivers by village council area and municipal ward.

The distribution of houses in close proximity to rivers was mapped by VCA and MW and is shown in Figure 2.

### Dengue incidence per village council area and municipal ward

**FIGURE 3:**
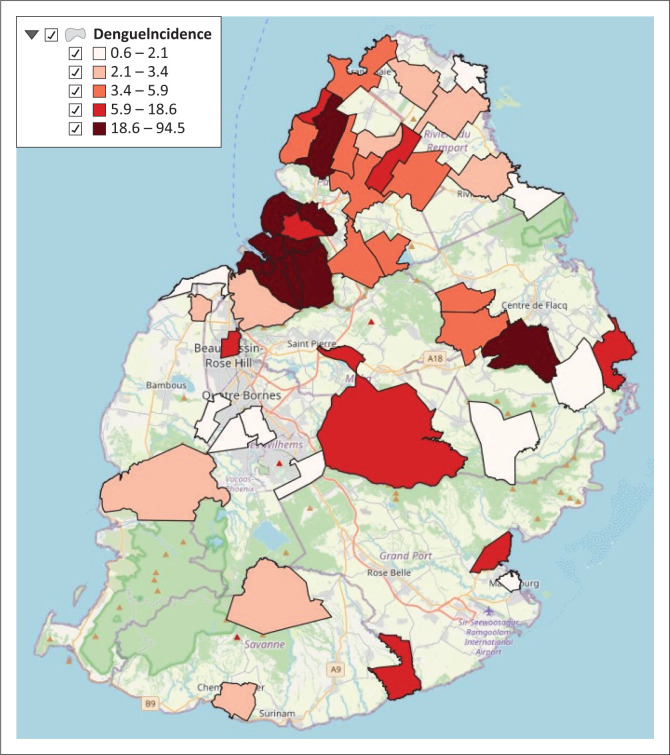
Distribution of dengue incidence (per 10 000 of population).

The cumulative dengue incidence (per 10 000 of population) was calculated for the period 2009 to 2023 and is shown in Figure 3. The highest dengue incidence was in MWs of the capital city of Port-Louis.

### Multiple regression analysis

A multiple regression analysis was carried out with dengue incidence per VCA and MW as the dependent variable and population density, the number of houses in close proximity to rivers, the RDI, the maximum rainfall and the minimum mean temperature as independent variables (Goorah 2024). The *R*-squared of 0.41 explained 41% of the variance as shown in Table 2. The model fit was statistically significant. The *F*-statistics was 5.69 (*p* < 0.001) as shown in Table 3.

**TABLE 2:** Regression statistics.

Regression statistics	Results
Multiple *R*	0.64
*R* square	0.41
Adjusted *R* square	0.34
Standard error	19.03
Observations	47

**TABLE 3:** Analysis of variance.

ANOVA	*df*	SS	MS	*F*	Significance *F*
Regression	5	10294.87	2058.97	5.69	0.00045
Residual	41	14843.56	362.04	-	-
Total	46	25138.42	-	-	-

ANOVA, analysis of variance; df, degree of freedom; SS, Sum of Squares; MS, Mean Square.

The results suggest that the proximity of houses close to rivers has a significant positive effect on dengue incidence (*p* = 0.03) implying that VCAs and MWs with more houses close to rivers had a higher dengue incidence. The RDI has a significant negative effect on dengue incidence (*p* = 0.01) implying that the more developed VCAs and MWs had lower dengue incidences. The population density, mean lowest temperature and maximum rainfall have no significant effect on dengue incidence per VCA and MW (*p* > 0.05) (Goorah 2024). This is shown in Table 4.

**TABLE 4:** Coefficients.

Variables	Coefficients	Standard error	*t* Stat	*p*	Lower 95%	Upper 95%	Lower 95%	Upper 95%
Intercept	131.94	136.68	0.97	0.34	144.10	407.97	144.10	407.97
POPDENSITY	0.00	0.00	1.22	0.23	0.00	0.01	0.00	0.01
RDI	**−201.05**	**76.20**	**−2.64**	**0.01**	**−354.94**	**−47.16**	**−354.94**	**−47.16**
RIVERPROX	**0.03**	**0.01**	**2.31**	**0.03**	**0.00**	**0.06**	**0.00**	**0.06**
MEANLOWTEM	1.40	4.67	0.30	0.77	−8.02	10.82	−8.02	10.82
MAXRAINFALL	0.00	0.01	−0.53	0.60	−0.02	0.01	−0.02	0.01

Note: The bold values are the statistically significant results with *p* < 0.05.

RDI, Relative Development Index; Stat, statistic.

In conclusion, dengue incidence = 131.94−201.05 × RDI + 0.03 × RIVERPROX

Relative Development Index is significant and negatively related to dengue incidence. RIVERPROX (number of houses situated close to rivers) is significant and positively related to dengue incidence. MEANLOWTEM (Mean Low Temperature) (*p* = 0.77), POPDENSITY (Population Density) and MAXRAINFALL (Maximum Rainfall) (with high *p*-values) are excluded because they are not statistically significant predictors.

The *R*-squared of 0.41 explained only 41% of the variance. Dengue incidence depends on many factors namely environmental, human behavioural and social patterns, mosquito biological factors, immunity levels in the population among others. Hence, the complexity of dengue incidence cannot be captured in a simple regression model with few predictors. More studies are required to explain dengue transmission more fully.

## Discussion

In this study, we have employed mapping techniques to display the spatial distribution of dengue. Geographic Information System is a powerful software that can be used to map, visualise, and analyse spatial data, making it practical for tracking the propagation of MBDs. By integrating GIS into our analysis, we can visualise locations and patterns of transmission, and analyse interactions between different driving forces. In addition, the use of open-source software is of value in a developing country with resource constraints like Mauritius.

The growing importance of real-world data derived from sources other than traditional sources is emerging. In addition to inputs from official sources, we have incorporated observational data gathered during recent outbreaks from social media platforms, local experts, press reports, radio and television broadcasts, and compiled these to obtain a measure of disease incidence. Real-world data based on real-world sources such as insights from social media platforms is increasingly being utilised in clinical research and in public health (Liu & Panagiotakos 2022). In this study, the practical use of real-world data from non-traditional sources has enriched our understanding of outbreaks.

The present study has a few limitations. Data regarding the precise locations of infected people or infection at household level are not accessible. This limitation constrains the granularity of data available for a more detailed study. In the absence of fine-scaled data, we have obtained figures at MW and VCA levels, and this has an impact on the accuracy of case locations and the precision with which outbreaks can be analysed.

We have observed a change in the spectrum of MBDs in Mauritius over time. Disease burden has shifted from traditional diseases like malaria to emerging infections such as chikungunya and dengue fever. This is similar to worldwide trends and has implications for public health preparedness.

Historically, malaria was the dominant MBD in the island since 1865. It became endemic and was responsible for severe outbreaks in the last two centuries. Important public health efforts were dedicated to its control and elimination. A turning point was reached in 1973 when malaria was officially eradicated from Mauritius. This was a significant achievement for the country and showed that MBDs can be eliminated. Subsequently, there were some limited outbreaks because of the introduction of imported cases, but they remained contained with effective public health measures.

In recent times, Mauritius has been confronted with emerging infectious disease challenges with the first introduction of chikungunya in the island in 2005 followed by a massive outbreak in 2006. Unlike malaria, chikungunya has so far failed to establish itself as an endemic disease. Despite the occurrence of imported cases annually, the country has not witnessed any chikungunya outbreaks since 2006.

However, a new MBD threat has manifested with the re-introduction of dengue fever in 2009 and recurrent outbreaks in 2014, 2015, 2016, 2019, 2020 and 2023. Trends suggest that this disease has the potential to become endemic resulting in the continuous occurrence of outbreaks. It is to be noted that the disease had mainly been caused by a single serotype until 2019 when there was the confirmed co-circulation of two serotypes, namely DENV-1 and DENV-2.

A systematic review carried out in the neighbouring island of Reunion has shown that dengue cases have increased considerably in number and in severity for the years 2018–2021 with annual epidemics and the co-circulation of several serotypes (Hafsia et al. 2022). Moreover, there is low-level circulation of dengue viruses in the interepidemic periods during the winter months.

The authors hypothesised that the shift to endemicity in Reunion is because of the following factors: an increase in serologically naïve young population, a degradation of the natural environment, increased use and disposal of manufactured containers and warmer temperatures since 2017. These favour an abundance of the *Aedes albopictus* mosquito vector. Increased international mobility of people may also be a contributory factor. A similar trend has been observed in another French island of the Indian Ocean, namely Mayotte (Hafsia et al. 2022). It can be surmised that Mauritius will also follow a similar progression with increased outbreaks, co-circulation of serotypes and endemic dengue.

The shift to emerging arbovirus infections also reflects global trends. Indeed, the dominance of malaria (transmitted by *Anopheles* species) in sub-Saharan Africa, South America, Central America and the Caribbean is giving way to diseases caused by arboviruses (transmitted by *Aedes* species), such as dengue and chikungunya as well as Zika in the Americas (Mordecai et al. 2020).

This is inferred to be because of climate change resulting in warmer temperatures, which favour the abundances of *Aedes* species over *Anopheles* species. According to a recent study, the authors have observed that arbovirus transmission peaks at 4 °C higher than malarial transmission (Mordecai et al. 2020). Thus, it is also important to consider changes in MBDs in the light of global warming for effective public health vigilance.

### Main driving forces of mosquito-borne diseases

The main results of this study show that RDI and the proximity of houses to rivers contribute to the early stages of an MBD outbreak. These findings are useful as they allow the development of targeted control strategies. It should be emphasised that other potential factors that could promote the propagation of disease such as the mobility of people and people’s behavioural and preventive habits that affect mosquito proliferation and human–mosquito interactions and bites were not explored at this stage.

### Climatic variables and mosquito-borne diseases

Climatic variables such as rainfall and temperature were studied. The minimum mean temperature and maximum rainfall were not statistically observed to influence disease incidence at VCA and MW levels. As localised climate conditions within a relatively small area may differ from the overall climate of a larger region and also vary from LTM values, we cannot exclude the contribution of temperature and rainfall in outbreak initiation. A previous study has shown that the 2006 chikungunya outbreak was linked to higher than mean rainfall (Ramchurn et al. 2008).

### Socioeconomic determinants and mosquito-borne diseases

Evidence presented in this study suggests that RDI, and by extension socioeconomic deprivation and poverty, increases the vulnerability to MBDs in Mauritius. As previously described, RDI is a composite index which comprises an assessment of physical housing structure, piped water supply and sanitation as well as the literacy level and occupational status of inhabitants. It is to be noted that RDI does not include waste management and garbage collection. Since RDI englobes several dimensions of deprivation, the primary factor influencing susceptibility to MBD is yet to be identified.

It is widely assumed that MBDs are diseases of poverty. Nevertheless, studies exploring the association of socioeconomic determinants and MBDs have reported inconsistent relationships (Mulligan et al. 2015; Whiteman et al. 2020). Poverty is assessed by different socioeconomic indicators in the published literature namely level of income, socioeconomic class and occupational status, literacy rate, housing infrastructure, garbage collection and availability of water among others. This makes it difficult to compare findings and to extract the most relevant indicator of poverty linked with MBDs.

One study reported that income and physical housing infrastructure were more closely associated with dengue than other poverty indicators (Mulligan et al. 2015). Another study reported that the effect of socioeconomic determinants was location specific, and both situationally and culturally dependent. In addition, consistent effects were obscured by the pervasiveness of mosquitoes and lack of viral immunity across all socioeconomic gradients (Whiteman et al. 2020).

In the context of our study, the importance of socioeconomic determinants highlights the question of whether vector control measures should be adapted to different socioeconomic groups for optimal results.

### Proximity to rivers and mosquito-borne diseases

The contribution of houses close to rivers in promoting the incidence of dengue is an interesting finding. Proximity to water bodies that act as breeding grounds and larval habitats is known to increase mosquito abundance. In addition, the rivers in Mauritius are mostly slow-moving rivers with thick vegetation comprising grass, shrubs and trees at the edges, which promote mosquito proliferation. This observation was also previously described by studies carried out by Ross as mentioned earlier (Ross 1908). Our results have confirmed statistically that zones with more houses close to rivers have a higher incidence of MBDs.

In this study, we have investigated the evolution of MBDs in Mauritius using historical and real-time data, and we have visualised geographical vulnerability using the open-source software QGIS. Using statistical analysis, we have identified factors namely RDI, proximity of houses close to rivers and population density as predictors of MBDs. The minimum mean temperature and maximum rainfall were not statistically observed to influence disease incidence at VCA and MW levels. However, it is possible that they are relevant at finer scale levels, which we were unable to study because of the absence of meteorological data at locality levels. It is thus important to have climatic data at locality levels available in the public domain so that further analysis can be carried out. Our findings have implications for public health measures, which can be designed to target these contributory factors.
